# Living with a pacemaker: patient-reported outcome of a pacemaker system

**DOI:** 10.1186/s12872-018-0849-6

**Published:** 2018-06-04

**Authors:** Peter Magnusson, Per Liv

**Affiliations:** 1 Cardiology Research Unit, Department of Medicine, Karolinska Institutet, Karolinska University Hospital/Solna, SE-171 76 Stockholm, Sweden; 2 Centre for Research and Development, Uppsala University/Region Gävleborg, Gävle, Sweden

**Keywords:** Arrhythmia, Complication, Experience, Pacemaker, Pocket

## Abstract

**Background:**

The aim of this study was to assess among pacemaker patients their overall satisfaction with the pacemaker system, pain, soreness/discomfort, cosmetic results, restrictions due to impaired movement of the shoulder/arm/chest, related sleep disturbances, and concern about possible device malfunction.

**Methods:**

The seven-item questionnaire was mailed to patients from a single center who had a pacemaker implant or replacement between 2006 and 2016. A higher score indicated worse outcome on a visual analog scale (VAS) of 0–100 mm.

**Results:**

The response rate was 75.5% and 342 questionniares were analyzed. Median age of respondents was 77.6 years and 57.0% were males. In total, 65 complications requiring surgery (10 pocket corrections (2.9%), 5 in females) occurred during a median follow-up of 5.6 years.The distribution of the primary outcome had a median score of 5 while the 75th percentile was 13. Cosmetic appearance was significantly associated with reoperation (but not other variables). Overall scores for men and women were 5 vs. 6, respectively, which achieved significance (*p* = 0.042). Median ratings of pain, soreness/discomfort, cosmetic appearance, range of motion, sleep, and concern about device malfunction were all ≤5. Females reported worse outcomes for all questions, except for cosmetic results and concern about malfunction.

**Conclusions:**

The vast majority of patients report excellent overall satisfaction with the pacemaker system, and are not affected by pain, soreness/discomfort, or concern about device malfunction. They also reported favourable outcomes with respect to cosmetic results, shoulder movement, and sleep. However, some patients underwent a surgical correction of the pacemaker pocket.

## Background

A permanent pacemaker is indicated in patients with bradycardia, i.e. second- or third-degree atrioventricular block, significant sinus node dysfunction, tachycardia-bradycardia syndrome, bundle branch block with a history of syncope, and, in specific circumstances, in various disease states, according to guidelines [1]. In symptomatic patients with heart failure, with an ejection fraction ≤40% and bundle branch block despite optimal medical therapy, cardiac resynchronization therapy (CRT) is indicated [1]. The implantation incidence in Western Europe is 938 bradycardia pacemakers and 140 CRT devices per million annually [2].

A transvenous pacemaker system consists of one (VVI or AAI) or two leads (DDD) fixated in the right side of the heart; a CRT devices adds a special lead on the left side. The lead(s) is plugged into a pacemaker device (50 × 50 mm and 5-7 mm thick and weight 20-30 g), which is inserted beneath the collarbone, typically on the left side. Perioperative complications can occur during vascular access (pneumothorax, arterial puncture, and nerve plexus injury) and during lead fixation in the myocardial wall (perforation, tricuspid valve damage, and sustained arrhythmias) [3, 4]. Postoperative and long-term complications requiring surgical revision include infections, lead malfunction due to oversensing or mechanical failure, technical device failure, and discomfort with the device system [5]. In many countries, these complications are reported in a national register e.g. the Swedish Pacemaker Registry and the Danish Pacemaker Register [6, 7]. In a validation study of the latter registry, 9.5% of cardiac implantable device patients were affected by complications after half a year [8]. However, the patient-reported experience, specifically with regard to the pacemaker system, is not completely understood [9]. Furhermore, generic questionnaires on health-related quality of life lack disease specificity [10]. Therefore we developed a questionnaire to assess overall satisfaction with the pacemaker system (primary outcome), and to assess secondary outcomes of pain, soreness/discomfort, cosmetic results, restrictions of movement impairment of the shoulder/arm/chest, sleep disturbances related to the pacemaker generator, and concern about device malfunction. We aimed to address these research questions in a cross-sectional cohort study in an unselected population of pacemaker patients with various duration since first implantation.

## Methods

### Setting

The records of all patients ≥18 years who recieved an initial pacemaker or a replacement pacemaker at Gävle Hospital, Region Gävleborg between December 2006 and December 2016 were extracted from the electronical system Provisio™.

We excluded patients with a history of primary a CRT implant and/or an implantable defibrillator. We used search codes FPE00, FPE10, FPE20, and FPE40 according to the International Code of Disease and Classification [11], which have not changed during the study period.

### Data collection and power analysis

The search yielded 2950 patients. A power analysis was performed using Monte-Carlo simulations from a hypothetical outcome of the primary outcome: log-normal distribution, truncated at 10 mm with a log-scale mean score of 1.6 and a log-scale standard deviation of 1. The simulations showed that 400 patients would under the described circumstances be sufficient to estimate the median VAS score of with an expected 95% confidence interval width of 7 mm, as estimated from BCa non-parametric bootstrapping. This was deemed to be a sufficient precision.

To compensate for non-responses, one fifth of the patients (590 out of 2950) were randomly selected using a computer script written in *R* and manually entered into the daily updated census register to ensure that we included only living patients [12]. After removal of duplicates, the final sample consisted of 453 patients, who were mailed the questionnaire.

In early January of 2017, the questionnaire was sent by regular mail together with information about the study, an informed consent form, and a return envelope. A reminder was sent 6 weeks later and a final reminder another 6–8 weeks thereafter. A phone call preceded the last reminder. In addition, a phone call was made to patients who returned incomplete questionnaires.

### The questionnaire

**Fig. 1:**
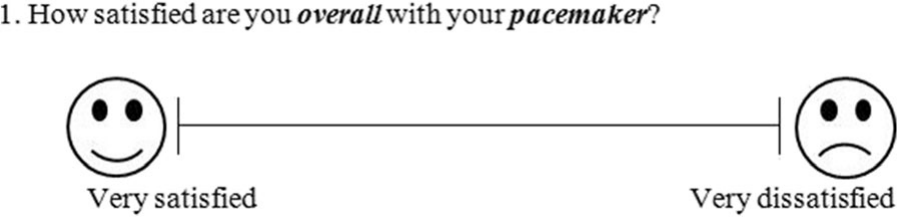
Question 1 in the questionnaire. A higher score indicates worse outcome

The questionnaire consisted of seven questions, to be answered on a 100 mm visual analog scale (VAS) with wording and pictures at each end (see example Fig. 1). A higher score indicates worse outcome.

The questions appear below with the words at either extreme on the VAS shown in parentheses.

1. How satisfied are you **overall** with your **pacemaker**? (Very satisfied, Very dissatisfied).
2. How much **pain** related to your **pacemaker** do you experience? (No pain, Considerable pain).
3. How much are you bothered by **sorenesss/discomfort** from the **pacemaker**? (No soreness/discomfort, Considerable soreness/discomfort).
4. How do you feel about the **cosmetic** appearance of your **pacemaker**? (Very Good, Very bad).
5. Do you experience any **restrictions of movement** of shoulder/arm/chest related to your **pacemaker**? (No restriction of movement, Considerable restriction of movement).
6. How would you rate any **sleep disturbances** related to your **pacemaker generator**? (No sleep disturbance, Considerable sleep disturbance).
7. How much **concern** do you feel that your **pacemaker** will **stop working** or **malfunction**? (No concern, Always concerned).

The questionnaire was developed by the authors and face validity was addressed by input from physicians and nurses involved in the follow-up of pacemaker patients. Laypeople were consulted to assure that the form was clearly understood and easy to follow. As generic quality-of-life instruments could not capture the specific patient-reported outcomes this paper aimed to address and as no suitable disease-specific instrument could be found in the literature, we developed a new questionnaire based on clinical experience. This new questionnaire has not been evaluated with regards to reliability or validity, which we recognize as a limitation.

### Other variables

In addition to the questionnaire, we asked patients to fill in their current medications, which were categorized as follows: sleeping pills (zolpidem, propiomazin) including other benzodiazepines, acetaminophen (paracetamol), selective serotonin reuptake inhibitors, opioids, and corticosteroids. Complications were defined as those requiring surgery involving the pacemaker system, i.e. opening of the pacemaker pocket. Information on complications was obtained from medical records and registry data.

### Statistics

Numeric data were described as frequencies, percentages, medians (interquartile range [IQR]), means (standard deviation [SD]), and percentiles. All VAS-scores on the questionnaire were reported in millimeters (mm). Tests of differences in VAS results between subgroups were performed using the Mann-Whitney U-test. Associations between continuous variables were assessed using Spearman’s correlation coefficient. Possible differences in proportion of response rates between males and females were tested using Fisher’s exact test. The choice of using non-parametric statistics was made based on non-normality of all measured variables, as seen from graphically examination of data. All statistical tests were two-sided with a significance level of 0.05. The database in Excel 2010 (Microsoft Corporation, Redmond, WA) was imported for analyses using *R (*R Core Team*, 2015).*

### Ethics

The Regional Ethical Committee in Uppsala approved the study (protocol number 2016/478).

## Results

A total of 342 questionniares were analyzed. Ages ranged from 30 to 100 years, with a median age of 77.6 years (interquartile range [IQR]: 70–84) and the mean age was 75.9 years (standard deviation [SD] 12.0). There were more males (*n* = 195; 57.0%) than females (*n* = 147; 43%). Patients had DDD (76.6%), single-chamber (17.0%), or CRT (6.4%) pacemakers. During the median time since primary pacemaker implant of 5.6 years (mean 6.5 years, SD 5.1), 65 complications requiring surgical intervention occurred, mainly lead-related malfunction or perforation. One patient had two complications. Notably, 10 patients (2.9%), of whom 5 were females, underwent pacemaker pocket revision, i.e. a correction. Median time since last device surgery was 4.3 years (IQR 1.73–8.1 years). In our study, over a median time since primary implant of 5.6 years, patients underwent device replacement either before battery depletion (*n* = 104, 30.4%) or due to complications (*n* = 51, 14.9%). The pharmacological regimens that could influence the patient’s pacemaker experience were mainly sleeping pills including benzodiazepines (*n* = 28; 7.3%), acetaminophen (paracetamol) (*n* = 18; 5.3%), selective serotonin reuptake inhibitors (n = 18; 5.3%), opioids (*n* = 8; 2.3%), and corticosteroids (*n* = 4; 1.2%). More than half of the patients (59.4%) were overweight (body mass index [BMI] > 25) at the time they completed the questionnaire. Median BMI was 26.0 kg/m^2^ (IQR 24.6–29.3) and only 3.3% reported BMI < 20 kg/m^2^. Patient characteristics are summarized in Table 1.

**Table 1:** Characteristics of 342 pacemaker patients

Variable	n
Number of patients	342
Median age (years)	77.6 (IQR 70.4–84.2)
Males	195 (57.0%)
Pacemaker type	
DDD	262 (76.6%)
VVI	51 (14.9%)
AAI	7 (2%)
CRT-P	22 (6.4%)
Number of procedures	
1	229 (67.0%)
2	67 (19.6%)
3	29 (8.5%)
4	15 (4.4%)
5	1 (0.3%)
6	1 (0.3%)
Complications requiring reintervention	
Lead malfunction	40 (11.7%)
Pocket correction	10 (2.9%)
Perforation	5 (1.5%)
Extraction	9 (2.6%)
Exploration unipolar lead	1 (0.3%)
Body-mass index (kg/m^2^)	
< 20	11 (3.3%)
20–25	119 (35.7%)
25–30	130 (39%)
> 30	73 (21.9%)

### Analysis of non-response

The response rate of the questionnaire was 75.5%. Reasons for non-response were as follows: dementia (*n* = 10), no registered postal address/emigration (*n* = 6), or unknown (*n* = 95). Fisher’s exact test revealed no stastically significant difference in response rate with regard to sex; 78.0% among males returned the questionnaire and females 72.4% (*p* = 0.188). Non-responders were significantly older than responders (median age: 82.4 years vs 77.6 years, *p* = 0.018).

### Primary outcome

**Fig. 2:**
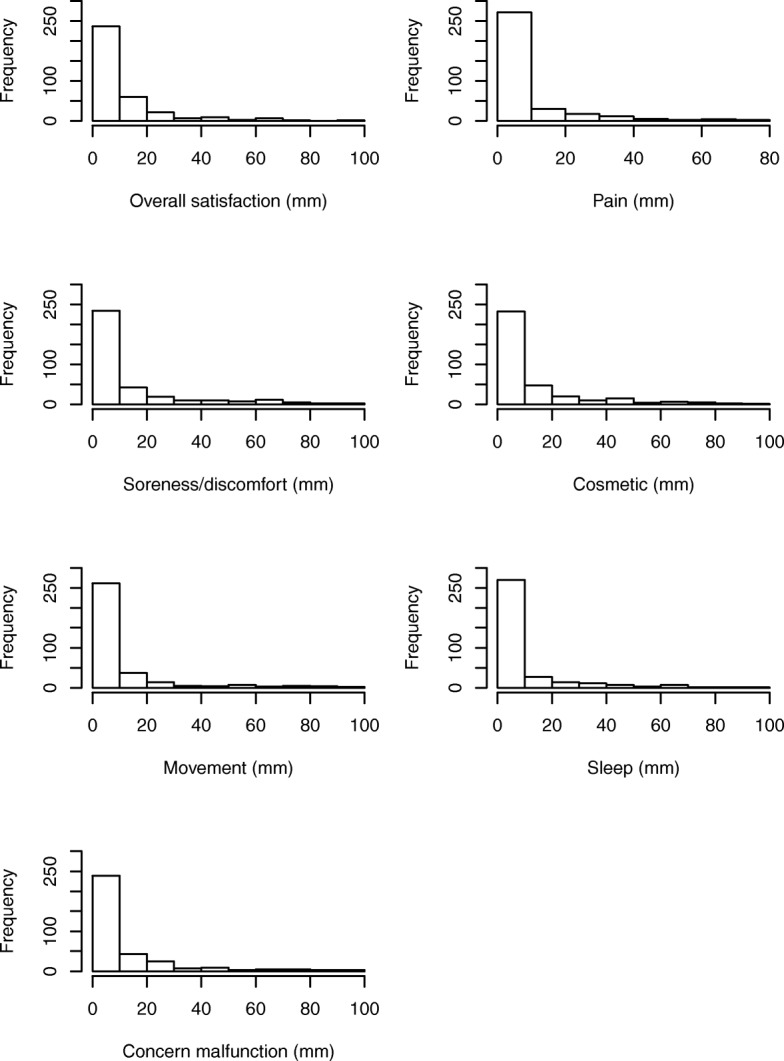
Histogram of frequency and outcome of pacemaker-patient reporting on a 100 mm scale. A higher score indicates worse outcome

As for the ratings of each seven questions, the distribution of the primary outcome *overall satisfaction* was heavily skewed to the right (Fig. 2). Median score was 5 while the 75th percentile was 13 and the 95th percentile 44.9.

Men and women had similar median scores (5 vs. 6, respectively) but the Mann-Whitney U test revealed statistical significance (*p* = 0.042) driven by differences in the higher percentiles (75th percentile:15.5 vs 11.5; 95th percentile: 54.2 vs 30.3). Patients who underwent reoperation did not report a significantly different outcome in overall satisfaction compared to those who did not undergo a revision (*p* = 0.14).

### Secondary outcomes

Median ratings of *pain, soreness/discomfort, cosmetic results, movement*, *sleep*, and *concern about device malfunction* were all ≤5. The 75th percentiles ranged from 8 (*pain*) to 16 (*cosmetic results*) and the 95th percentile ranged from 38 (*pain*) to 54 (*movement*), see Tables 2 and 3. Statistically significant differences between males and females were found for all questions, except for *cosmetic results* and *concern about device malfunction*. Reoperation was significantly associated withworse outcome for *cosmetic results,* but not for the other variables.

**Table 2:** Percentile distribution and mean values (standard deviations) of responses on VAS-scale (mm)

Question	5%	25%	50%	75%	95%	Mean(SD)
Overall satisfaction	0	2	5	13	44.9	10.3 (14.1)
Pain	0	1	3	8	38	8.3 (13.1)
Soreness/discomfort	0	2	4	14	62	13 (19.5)
Cosmetic results	0	2	5	15.8	50.9	12.4 (17.9)
Movement	0	1	4	10	53.9	10.3 (17.8)
Sleep	0	2	4	9	44.8	9.2 (14.8)
Concern about malfunction	0	2	4	13	52.9	11.7 (18.4)

**Table 3:** Median and interquartile range (IQR) with regard to sex and reoperation (*p*-value using Mann-Whitney U-test)

Question	Females, median (IQR)	Males, median (IQR)	*p*-value	No reop, median (IQR)	Reoperation, median (IQR)	*p*-value
Overall satisfaction	6 (3–15.5)	5 (2–11.5)	0.042	6 (2–14)	4 (2–11)	0.142
Pain	4 (2–11)	3 (1–7)	0.025	3 (2–9)	3 (1–8)	0.612
Soreness/discomfort	6 (2–20)	4 (1–11)	0.002	4 (2–14)	5 (2–15)	0.568
Cosmetic results	5 (2–17)	4 (2–14)	0.201	4 (2–13)	7 (2–21)	0.043
Movement	4 (2–13)	3 (1–8)	0.079	4 (1–10)	3 (1–8)	0.958
Sleep	5 (2–11.5)	3 (1–7)	0.009	4 (2–8)	3 (1–9)	0.595
Concern about malfunction	5 (2–13.5)	4 (1–12)	0.12	5 (2–13)	3 (2–11)	0.253

### Correlations

None of the seven outcome scores correlated significantly to *patient age at the time of evaluation* (Spearman’s rho ranging between 0.00 and 0.16, all *p*-values ≥0.355), *time since the first implantation* (Spearman’s rho between - 0.09 and − 0.01, all *p* > 0.12) or *BMI* (Spearman’s rho between - 0.10 and 0.00, all *p* > 0.48). However, a longer *time since last pacemaker surgery* was significantly correlated to better outcomes for all questions (overall satisfaction rho = − 0.26, *p* < 0.001; pain rho = − 0.21, p < 0.001; Soreness/discomfort rho = − 0.16, *p* = 0.01; cosmetic rho = − 0.16, p = 0.01; movement rho = − 0.22, p = 0.01; sleep rho = − 0.19, p = 0.01; concern malfunction rho = − 0.09, *p* = 0.04).

## Discussion

Pacemaker implantation typically results in lifelong therapy. From a healthcare perspective, complications related to pacemaker implantation may require surgical interventions as well as extra follow-up visits, either in-clinic or remotely by using home-monitoring. Notably, 10 patients complained about the device position and underwent surgical intervention to reposition the pacemaker. Clinicians may hesitate before making the decision to surgically correct the pacemaker pocket, because it increases the risk of infection and lead damage, which may necessitate lead extractions that involve serious risks and expense [13–16].

### Most patients report overall satisfaction

In the present study, the vast majority claimed very high *overall satisfaction* and further analyses of secondary outcomes confirmed this. Nevertheless, a non-neglible proportion of the patients reported complaints which did not result in surgical revisions because they were not addressed clinically. It is not clear from our study why some patient complaints were not identified during their follow-up.

### Females more often report pain, soreness/discomfort, and sleep disturbances

We found statistically significant differences in reported *overall satisfaction, pain soreness/discomfort* and *sleep* between the sexes, where females more frequently reported worse outcome than males. Since females are generally smaller,the device may more readily impede arm movement. Interestingly, there were few patients who reported cosmetic problems and there was no difference between males and females in the assessment of cosmetic results. Although the study population was 57% men, among the patients who underwent surgical revision to correct pacemaker position in the pocket, 50% were women (*n* = 5).

### Age, BMI, reoperation

Interestingly, neither age nor BMI seemed to affect the patient-reported outcomes for our survey. The fact that re-operation was not associated with worse outcome is reassuring, but it still must be remembered that surgical revision increases the risk of infection and may require lead extraction. From the perspective of each individual patient, it is important to take every measure to avoid reoperation due to complications and consider the optimal technique for pacemaker and lead placement at the initial implantation [17, 18].

### Future perspectives

This study provides insights into the perceptions of pacemaker patients about their device therapy. Despite the increasing proportion of home-monitoring devices, which have improved patient safety and clinical logistics, it is still important to evaluate outcomes from a holistic and pacemaker-patient-centric viewpoint. The creation of the pocket during the initial implantation is crucial. There is an ongoing trial designed to address whether intramuscular implantation is superior to a subcutaneous pocket for initial pacemaker placement [19].

The introduction of a new leadless pacemaker (Micra™) may eliminate problems due to the pocket and leads. However, this device has limited availability, few indications, and may be cost prohibitive [20, 21]. Rechargeable pacemakers seem to offer the theoretical advantages of reducing device replacements, but there are no rechargeable devices currently on the market.

### Strengths and weaknesses

This study evaluates pacemaker therapy from the patient perspective, going beyond data in registries in order to address patient attitudes without the potential bias that could be introduced by interviewing patients during clinical follow-up. Many of our respondents were elderly and some suffered cognitive impairments and were not able to follow instructions, even though the questionnaire was short and straightforward. Nonresponders tended to be older than respondents, but males and females participated in the study to the same extent.In order to overcome the limitations of a cross-sectional study design, repeated outcome assessments at predefined follow-up periods are required. Psychological coping strategies, like adaptation, were not addressed. Finally, we would like to point out that multiple statistical tests were performed in our study, but no correction for mass significance has been made, which means that caution is warranted when interpreting individual tests. An alternative approach would have to perform such corrections to reduce the risk of statistical type 1-errors, but at the cost of an increased risk of type 2-errors.

## Conclusion

The vast majority of pacemaker patients report excellent overall satisfaction with their pacemaker system, including the absence of pain, no soreness/discomfort, acceptable cosmetic results, good shoulder movement, sound sleep, and no concerns about device malfunction. In 2.9% of the study population, a surgical procedure was need to correct device placement. Females report worse overall satisfaction, pain, soreness/discomfort, and more sleep problems related to the pacemaker.

## Abbreviations

BMI: body mass index
CRT: cardiac resynchronization therapy
IQR: interquartile range
SD: standard deviation
VAS: visual analog scale
